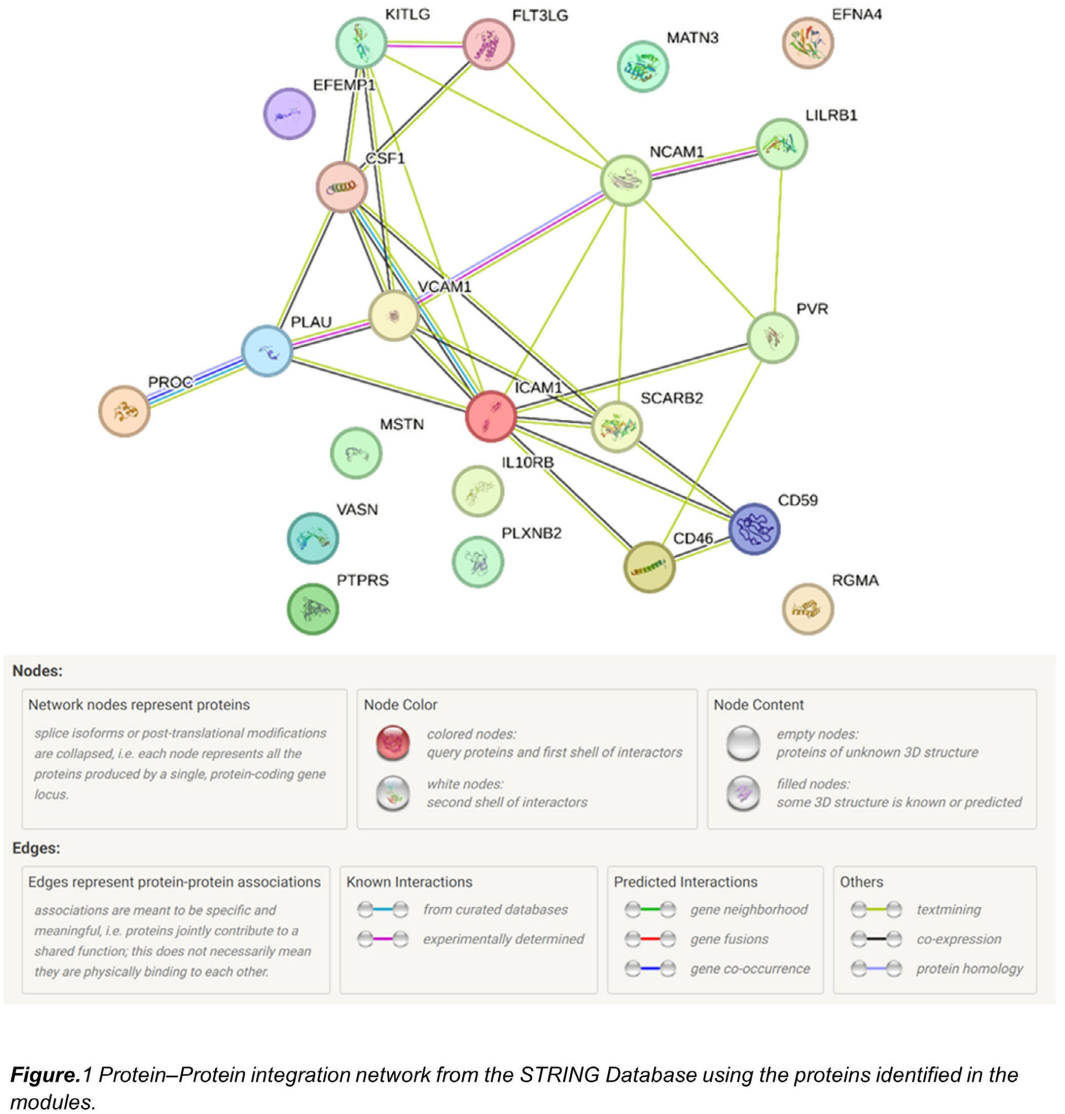# Proteomic Signature of The Cerebrospinal Fluid (CSF) in Preclinical and prodromal AD: A key role for adhesion proteins

**DOI:** 10.1002/alz.086918

**Published:** 2025-01-09

**Authors:** Reem M. Neal, zhiyi Yang, Ihab Hajjar

**Affiliations:** ^1^ UT Southwestern Medical Center, Dallas, TX USA; ^2^ Emory University, Atlanta, GA USA

## Abstract

**Background:**

Alzheimer's Disease (AD) proteomic studies have focused on understanding the pathophysiology of AD during the late stages of AD. However, recent studies have suggested that the preclinical stage of AD represents a golden window for intervention. Yet, little is known about the influence of the cerebrospinal fluid (CSF) molecular environment on the mechanisms underlying the pathogenesis of AD during the preclinical stage.

**Method:**

We performed targeted proteomics using Olink® platform that measured 276 proteins related to neuronal injury, endothelial function, and cardiometabolic pathways in samples from 354 participants recruited for the BSHARP study. Advanced bioinformatic pipeline for data reduction, protein‐protein interaction networks, and module development were used. Resultant Hub proteins that are associated with various clinical phenotypes and biological traits such as levels of CSF AD biomarkers (Aβ42, Tau, and pTau) and the Aβ42/Tau Ratio (TAR) were identified. We then ran the list of Hub proteins through the STRING database to identify critical proteins and performed a pathway enrichment analysis to understand their role in AD.

**Result:**

We compared CSF and plasma proteomic profiles between participants who have normal cognitive function and those who have based on TAR status. As a result, we identified critical proteins that are associated with AD clinical phenotypes and levels of CSF AD biomarkers such as Aβ42, Tau, and pTau (all p‐values <0.05). We also found a significant difference in levels of 6 of the critical genes based on TAR status (HGF, ICAM1, VCAM1, NRP1, NRP2, SCARB2, CCL3). Pathway enrichment of the critical proteins using Gene Ontology (GO) Biological Process identified a series of pathways related to cell adhesion, inflammation, and endothelial function as well as response to Amyloid (all p‐value <0.05) pathways which are known to be related to AD.

**Conclusion:**

Our findings reveal that adhesion and endothelial‐related pathways are highly associated with AD phenotypes, especially in the preclinical stage. This suggests a possible proteomic signature in the CSF of individuals with preclinical AD that is driven by adhesion molecules and contributes to the pathogenesis of AD. Future studies investigating this contribution might provide insight into the molecular mechanisms underlying AD.